# Parecoxib alleviates the inflammatory effect of leukocyte-rich platelet-rich plasma in normal rabbit tendons

**DOI:** 10.1186/s12891-020-03793-2

**Published:** 2020-12-10

**Authors:** Ming Zhou, Ning Wang, Gang Wang, Zishan Jia, Xiaolei Qi

**Affiliations:** Department of Rehabilitation Medicine, First Medical Center of Chinese PLA General Hospital, 28 Fuxing Road, Beijing, 100853 People’s Republic of China

**Keywords:** Platelet-rich plasma, Parecoxib, Nonsteroidal anti-inflammatory drugs, Tendinopathy

## Abstract

**Background:**

Platelet-rich plasma (PRP) is widely used to treat tendon injuries. Its therapeutic effect varies depending on the different cell components, and white blood cells (WBCs) may play an important role in this phenomenon. The purpose of this study was to evaluate how PRP with different concentrations of WBCs affect normal rabbit tendon and assess whether non-steroidal anti-inflammatory drugs (NSAIDs) can suppress the catabolic effects of WBCs.

**Methods:**

Sixteen adult New Zealand White rabbits were used. Blood samples were collected from each rabbit, and PRP was extracted following two different protocols to obtain leukocyte-poor PRP (LP-PRP) and leukocyte-rich PRP (LR-PRP). LP-PRP or LR-PRP was injected into the patellar tendon of each rabbit, while normal saline (NS) was injected as control. In LR-PRP + NSAID group, Parecoxib was administered after LR-PRP injection. For each group, 2 rabbits were euthanatized at day 5 and 14. The patellar tendons were collected and stained with hematoxylin and eosin. A semi-quantitative approach was used to assess the inflammatory response and tendon destruction based on the evaluation of the WBCs, vascularization, fiber structure, and fibrosis.

**Results:**

The LR-PRP group exhibited a higher total tendon score than the LP-PRP group at day 5 after PRP injection, but there was no significant difference between the two groups at day 14. For the NSAID group, the tendon score was lower than that of the LR-PRP group both at day 5 and 14.

**Conclusion:**

LR-PRP can promote a higher inflammatory response than LP-PRP in the normal rabbit patellar tendon, and this effect can be suppressed by NSAIDs.

## Background

Platelet-rich plasma (PRP) is characterized by a high concentration of platelets, and is prepared by centrifuging autologous whole blood samples collected with anticoagulants. After activation, PRP is rich in autologous growth factors, including the transforming growth factor (TGF-β1), platelet-derived growth factor (PDGF), and vascular endothelial growth factor (VEGF). These growth factors have the potential to stimulate the proliferation of fibroblasts (PDGF) [1], collagen synthesis (TGF-β) [2], differentiation of fibroblasts and myoblasts (IGF) [3], while VEGF and hepatocyte growth factor (HGF) have a synergic effect on angiogenesis [4]. For these reasons, the use of PRP for sports injury treatment has gradually increased [5–7]. Previous studies on PRP use are conflicting; some showed favorable results [8, 9], while others failed to reproduce these outcomes [10, 11]. Variations of the PRP extraction protocol used for different studies may play a role in the contradictory nature of these studies.

Some studies had shown negative outcomes after intramuscular PRP injection, including the infiltration of inflammatory cells, edema, and necrosis, during the early stage injection, followed by fibrosis in the chronic stage [12]. White blood cells (WBCs) produce various pro-inflammatory molecules, such as tumor necrosis factor–α (TNF-α) and interleukin-1β (IL-1β) [13], which are considered to be one of the causes of negative effects in sports injury treatments.

It has been reported that excessive inflammation is harmful for wound healing and tissue regeneration [14]. Parecoxib, a non-steroidal anti-inflammatory drug (NSAID), which is often used for postoperative pain management, selectively inhibits the activity of cyclooxygenase-2 (COX-2), and could be useful to control inflammation in the PRP-based treatment of tendon injuries [15, 16].

The aim of this study was to evaluate the inflammatory response caused by LR-PRP injection in normal rabbit tendon, coupled with Parecoxib administration. We hypothesized that LR-PRP will induce a higher inflammatory response and this effect can be suppressed by NSAID.

## Methods

### Animals

Sixteen adult New Zealand White rabbits (3–4 months old, 2.5–3.0 kg) were used in this study. The protocols for blood collection, tendon injection, and tendon collection from rabbits were approved by the Ethics Committee on the Care and Use of Animals of Chinese PLA General Hospital (Number 2016-X12–04), and all animals received humane care in strict accordance with the National Institutes of Health Guidelines.

### Blood collection and PRP extraction

From each rabbit, 27 ml of whole blood was collected through the central artery of ear using an 18-gauge needle that was reloaded with 3.8% sodium citrate. Rabbits ware infused with 27 ml normal saline (NS) to recover the blood volume. For cell counting, 0.5 ml of the whole blood sample was used; the remaining blood was mixed with 3.8% sodium citrate in a 9:1 ratio, and then centrifuged at 200 g for 20 min. In the LR-PRP group, the plasma on the top layer and the buffy coat (containing mainly WBCs) from the middle layer were transferred to a new tube, and centrifuged at 400 g for another 15 min. In the LP-PRP group, only the plasma layer was transferred into a new centrifuge tube. After the second centrifugation step, the lower 4.5 ml was used as LR-PRP and LP-PRP. From this volume, 0.5 ml was used for cell counting, and the remaining 4 ml was used for injections (2 ml/knee).

### Experimental procedures

**Fig. 1:**
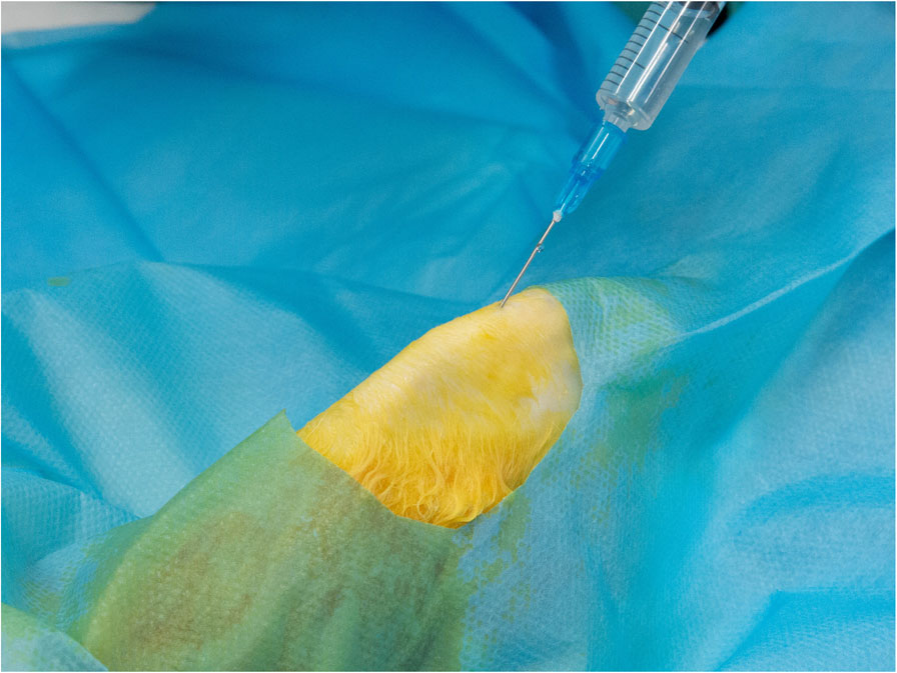
Normal saline was injected into patellar tendon. Total 2 ml media was injected at 10 different points. LP-PRP and LR-PRP was injected with the same method

Rabbits were anesthetized with ketamine (10 mg/kg) and xylazine (3 mg/kg). To ensure that all the tendon tissue was immersed with PRP or NS, 2 ml of solution was injected with a 25-gauge needle at 10 different points. **(**Fig. 1**)** Intra-tendon [17–19] and para-tendon [20] injection were both used in previous studies, but intra-tendon injection was adopted in our study to insure a full inflammation reaction. With the concern of tendon necrosis, a relatively low dose of media was injected and side effects such as necrosis was not observed in previous study [21].

A total of 16 healthy rabbits (32 patellar tendons) were used in this study. The animals were divided into 4 groups: control (NS-injected), LP-PRP, LR-PRP, and LR-PRP + NSAID. There were 4 animals (8 tendons) in each group. In control group, 2 ml NS was injected in each patellar tendon (4 animals, total 8 tendons). In LP-PRP group, 2 ml LP-PRP was injected in each patellar tendon (4 animals, total 8 tendons). In LR-PRP group and LR-PRP + NSAID group, 2 ml LR-PRP was injected in each patellar tendon (8 animals, total 16 tendons). Additionally, rabbits in LR-PRP + NSAID group were injected with Parecoxib (Pharmacia & Upjohn Company LLC, Kalamazoo, MI) 0.5 mg/kg intramuscularly every 24 h for 3 days. Rabbits were then caged individually and allowed to move freely.

### Sample collection and histological analysis

Animals were euthanatized 5 or 14 days after PRP administration through lethal dose injection of ketamine (10 mg/kg) and xylazine (3 mg/kg). From each group, 2 animals (4 tendons) were euthanized at day 5, and the other 2 at day 14. The entire patellar tendon was removed from the patellar to the tibia insertion site, fixed in 10% buffered formalin for 48 h, and embedded in paraffin. The samples were cut into 5 μm-thick sections, and stained with hematoxylin-eosin (HE) for histological analysis. All sections were analyzed under a microscope by two doctors in a blinded manner.

Sections were systematically assessed using the 40× objective, and 4 fields were selected within the treatment areas, which were characterized by tendon fiber destruction following PRP injection.

A semi-quantitative grading scheme [21] was used to evaluate the intensity of the inflammatory response based on the WBC number, vascularization, fiber structure, and fibrosis on a scale from 0 to 3. In this scheme, a high score suggests a high tendon inflammatory response.

### Statistical analysis

All results are represented as mean and standard deviation. Analysis of variance (ANOVA) was used for statistical analysis and the least significance difference (LSD) was used as post-hoc test. A difference of *p* < 0.05 was considered to be statistically significant.

## Results

### Analysis of autologous blood product

Compared to whole blood (WB), LP-PRP (2.06-fold) and LR-PRP (2.96-fold) had a higher platelet concentration; moreover, LR-PRP had a higher concentration of platelets and WBCs compared to LP-PRP. The results are shown in Table 1.

**Table 1:** Analysis of autologous blood products

	RBCx10^12^/L	WBCx10^9^/L	PLTx10^9^/L
WB	4.43 ± 0.44	2.71 ± 0.31	177.94 ± 49.46
LP-PRP	0.36 ± 0.21	1.70 ± 0.20	365.75 ± 21.85
LR-PRP	0.68 ± 0.13	4.87 ± 1.07	526.33 ± 52.54

*WB* Whole blood, *LP-PRP* Leukocyte-poor PRP, *LR-PRP* Leukocyte-rich PRP, *RBC* Red blood cell, *WBC* White blood cell, *PLT* Platelet

### Histological findings

**Fig. 2:**
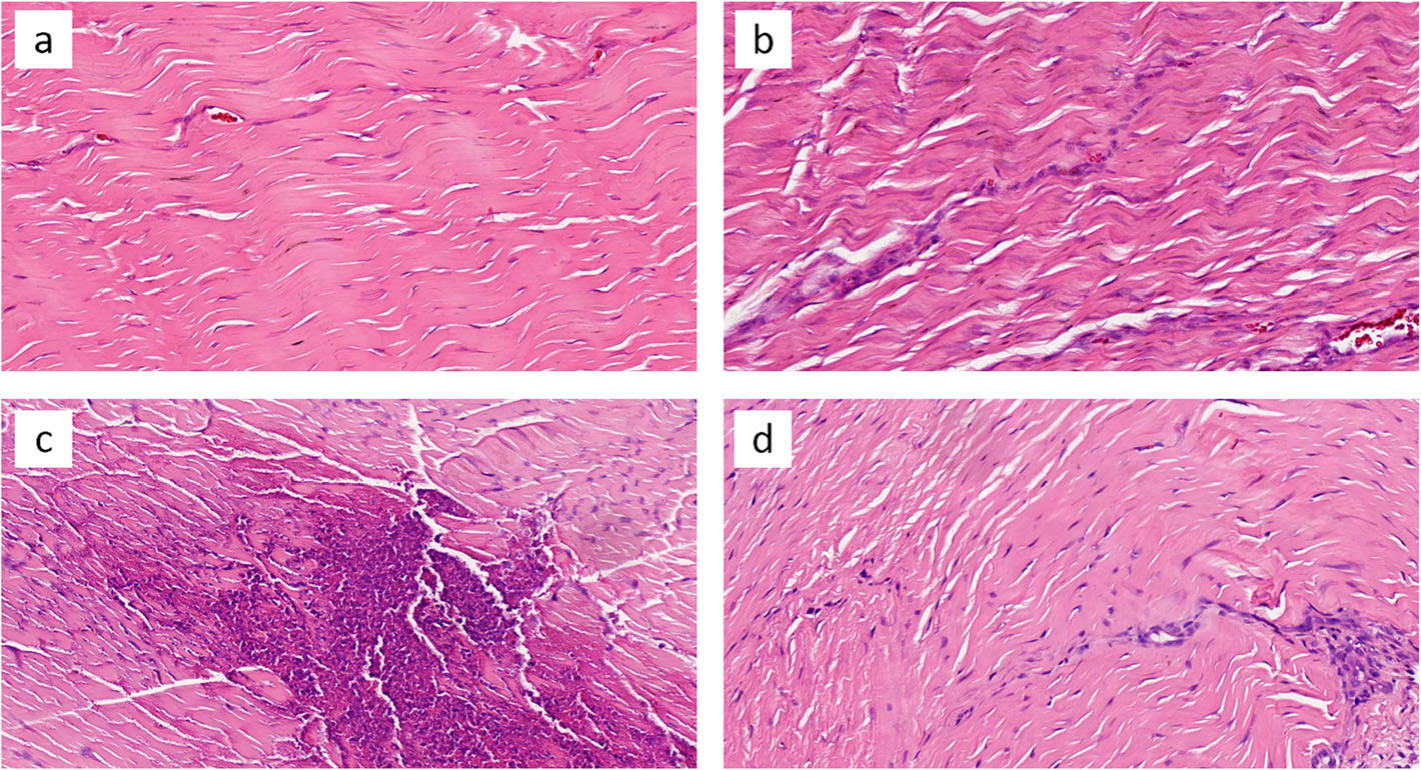
Representative sections of rabbit patellar tendon stained with Haematoxylin and Eosin, 5 days post-injection. Tendons treated with NS (**a**) showing regular arrangement of parallel collagen fibrils and cell numbers. While treated with LP-PRP (**b**) and LR-PRP (**c**), tendon showing a higher inflammation reaction with a higher cell infiltration and fiber deterioration. LR-PRP has the highest inflammation reaction, but after NSAIDs was add (**d**), the reaction is alleviated. (HE, 400 × magnification)

**Fig. 3:**
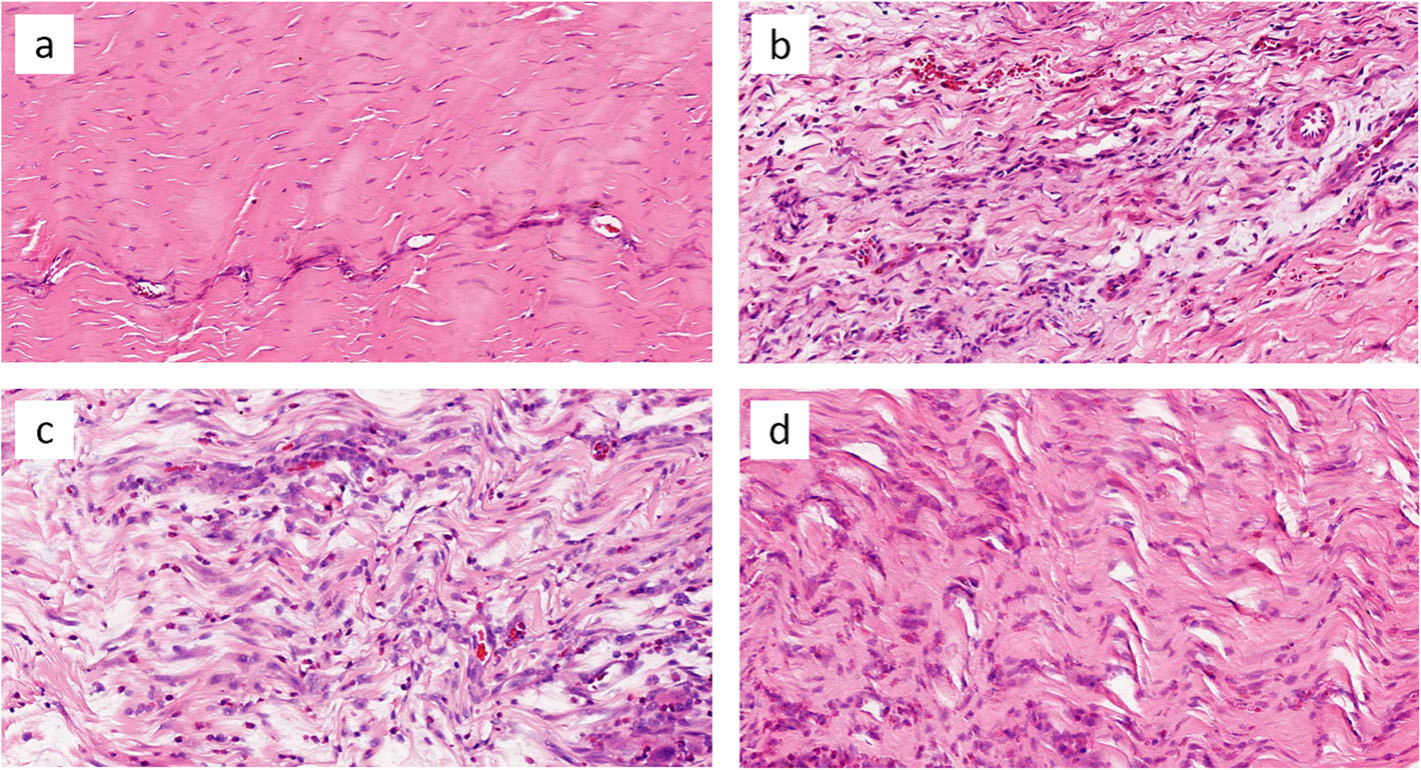
Representative sections of rabbit patellar tendon stained with Haematoxylin and Eosin, 14 days post-injection. Tendons treated with NS (**a**) showing relatively normal appearance. LP-PRP (**b**) and LR-PRP (**c**) has a comparable inflammation reaction and the cell infiltration is higher than 5 days. While in LR-PRP + NSAIDs group (**d**), the cell infiltration is lower than in LR-PRP group. (HE, 400 × magnification)

The LR-PRP group had a higher total tendon score than the LP-PRP group at day 5 post-injection (3.25, 95%CI 1.46 to 5.04, *p* = 0.002), but there was no significant difference between the two groups at day 14 (1.75, 95%CI − 0.21 to 3.71, *p* = 0.76). For the LR-PRP + NSAID group, the tendon score was lower than that of the LR-PRP group both at day 5 (2.25, 95%CI 0.46 to 4.04, *p* = 0.018) and day 14 (4.00, 95%CI 2.04 to 5.96, *p* = 0.001). The results are summarized in Table 2 and Table 3. The histological images are presented in Fig. 2 and Fig. 3.

**Table 2:** Histologic Data at day 5 post-injection

	WBCs count	Vascularization	Fiber Structure	Fibrosis	Total	*P* Value
NS	0	0.25 ± 0.5	0	0	0.25 ± 0.5	–
LP-PRP	0.75 ± 0.5	0.5 ± 0.58	0.5 ± 0.58	0	1.75 ± 1.5	0.093
LR-PRP	2.5 ± 0.58	0.75 ± 0.96	1.75 ± 0.96	0	5 ± 1.63	0.000
LR-PRP + NSAID	1.5 ± 0.58	0.5 ± 0.58	0.75 ± 0.5	0	2.75 ± 0.5	0.010

*P* values show total tendon score compared with NS group

**Table 3:** Histologic Data at day 14 post-injection

	WBCs count	Vascularization	Fiber Structure	Fibrosis	Total	*P* Value
NS	0.75 ± 0.5	0.75 ± 0.96	0.5 ± 0.58	0.25 ± 0.5	2.25 ± 1.7	–
LP-PRP	2.25 ± 0.5	1.5 ± 0.58	1.25 ± 0.5	1.25 ± 0.5	6.25 ± 0.5	0.001
LR-PRP	2.25 ± 0.5	1.75 ± 0.5	2.5 ± 0.58	1.5 ± 0.58	8 ± 1.15	0.000
LR-PRP + NSAID	1.75 ± 0.5	0.5 ± 0.58	1.25 ± 0.5	0.5 ± 0.58	4 ± 1.41	0.076

*P* values show total tendon score compared with NS group

## Discussion

The most important finding of the study was that LR-PRP, which contained a higher concentration of WBCs compared to LP-PRP, induced a higher inflammation reaction in normal rabbit tendon and this effect could be alleviated by administration of NSAIDs both in acute (5 days) and chronic (14 days) phase. This result confirm our hypothesis that LR-PRP induces a higher inflammatory response and this effect can be suppressed by NSAID.

The tendon is a tissue with low blood supply and has a low healing rate after injury. PRP contains many growth factors that have the ability to promote tendon stem cell differentiation [22] and tenocyte proliferation [23], and it is believed to have the potential to accelerate tendon healing. This has been widely proven by many basic and clinical studies [9, 24–26]. While most of the studies showed favorable results, some also showed contrasting results. In the study conducted by Harris et al. [12], PRP has been shown to induce an increased inflammatory response in normal rabbit muscle tissues, which includes the infiltration of lymphocytes and monocytes, edema, necrosis, and fibrosis which might be harmful to tendon healing.

The reasons for this phenomenon remain unclear. One probable reason is that various cell components and cytokines play a role in tissue destruction. Leukocytes, which secrete vast amounts of TNF-α and IL-1β, are considered to be the most important source of pro-inflammatory cytokines [13]. In a rat tendon injury model, Marsolais et al. [27] showed that inflammatory cells, such as granulocytes, ED1, and ED2 macrophages, rapidly accumulate in the injury site. These types of cells contain high amounts of proteases that can damage the tendon tissue and delay injury healing [28]. Dragoo et al. [21] compared acute inflammatory response of two commercial PRP products with different WBCs concentration. The result showed that PRP with higher WBCs concentration caused a greater inflammatory response in acute phase in tendon tissue.

Our study confirmed that LR-PRP, which had a higher concentration of WBCs, induced a higher tendon score than LP-PRP at day 5, thereby showing that WBCs cause an intense inflammatory reaction in the acute phase. This result was consistent with the previous study [21]. In contrast, after 14 days, there was no significant difference between the LP-PRP and LR-PRP groups, showing that the inflammation caused by different PRP injections tends to be comparable in the chronic phase. Furthermore, we investigated whether the inflammation effect could be suppressed by NSAIDs, in which way the damage activity caused by inflammation could be suppressed.

In most PRP extraction methods, higher platelet concentrations often correspond to higher concentrations of WBCs. A recent study by Castillo et al. [29] compared the products from 3 different commercially available PRP separation systems. The authors emphasize that there was a significant difference between the WBC concentration in products obtained with different systems, as well as different platelet and growth factor concentrations. Therefore, repressing the tissue damage activity of WBCs might decrease the inflammatory response and enhance tissue regeneration.

NSAIDs are often used to alleviate the inflammation in injured tissues by inhibiting the activity of cyclooxygenase. This results in a decrease in the synthesis of prostaglandins, which are pro-inflammatory molecules that have the ability to recruit leukocytes and other immune cells at the injury site [30]. A previous study indicated that NSAIDs can also block the adhesion molecules on WBCs, decrease their migratory abilities, and thus, reduce tissue damage [31]. NSAIDs are reported to have 30–50% sparing effect on morphine consumption to release pain postoperatively [32]. As a highly selective COX-2 inhibitor, which has a lower side effect compare to non-selective COX inhibitor, parecoxib is frequently used to treat many orthopedics diseases [33–35]. We selected parecoxib in the study also because it is an injectable COX-2 inhibitor, [35] which is helpful for accurate drug administration in animal experiment.

Regarding their impact on platelet activation and aggregation, the combinatorial use of NSAIDs and PRP to treat tissue injuries was discouraged in the past. In a recent study, Anitua et al [36] examined if the intake of NSAIDs could affect the properties of platelets. The results showed that none of the tested NSAIDs affected platelet activation. In addition, the concentration of molecules that play important roles in tissue regeneration, such as VEGF, PDGF-AB, and IGF-1, was not altered.

Our study showed that the inflammatory reaction caused by WBCs can be suppressed by NSAIDs both in the acute and chronic phases, as the tendon scores were lower in the LR-PRP + NSAID group compared to the LR-PRP group, both after 5 and 14 days. Although administered only during the first 3 days, NSAIDs impact both the time points. This effect might not have only been caused by suppressing the synthesis of prostaglandins, but also by reducing the WBC recruitment ability that might account for the long-term effect.

The potential limitation of this study was that, we only observed the cellular and histological effects of WBC-induced inflammation, and further studies were required to elucidate the underlying molecular mechanisms. As mentioned above, Anitua et al [36] has proved that the intake of NSAIDs does not alter the biological properties of platelets in vitro, including the cytokine concentration of inflammation, cell proliferation, angiogenesis and cell migration. For this reason, we did not set a LP-PRP group to clarify the effect of NSAIDs on platelet. This could be another limitation of our study, in a following step, we need to test the histological effect of NSAIDs on platelet. For the reason that there is no ideal chronic tendinopathy model, only healthy tendons were analyzed in our study, a method which also adopted by previous study [21]; therefore, future studies should also include injured tendons to better simulate the clinical situation. Finally, the sample size was relatively low in our study, 95% CI was provided to better describe the result. Improvement in blood collection technique in smaller animals will help to increase the sample size in future study.

## Conclusion

Our study confirms that LR-PRP can cause an increased inflammatory response in the normal rabbit patellar tendon that can be suppressed by NSAIDs. This shows that NSAIDs may be used to protect tendons from the pro-inflammatory activity of WBCs, which are present in PRP.

## Data Availability

The datasets used and analysed during the current study are available from the corresponding author on reasonable request.

## Abbreviations

PRP: Platelet-rich plasma
WBCs: White blood cells
NSAIDs: Non-steroidal anti-inflammatory drugs
LP-PRP: Leukocyte-poor PRP
LR-PRP: Leukocyte-rich PRP
NS: Normal saline
TGF-β: Transforming growth factor
PDGF: Platelet-derived growth factor
VEGF: Vascular endothelial growth factor
HGF: Hepatocyte growth factor
